# Developing metrics for nursing quality of care for low- and middle-income countries: a scoping review linked to stakeholder engagement

**DOI:** 10.1186/s12960-020-00470-2

**Published:** 2020-07-10

**Authors:** David Gathara, Mathias Zosi, George Serem, Jacinta Nzinga, Georgina A. V. Murphy, Debra Jackson, Sharon Brownie, Mike English

**Affiliations:** 1 KEMRI Wellcome Trust Research Programme, P.O Box 43640 00100, Nairobi, Kenya; 2 School of Nursing and Midwifery, Aga Khan University, P.O Box 39340 00623, Nairobi, Kenya; 3 Kenya Medical Training College, Kilifi Campus, Nairobi, Kenya; 4 Nuffield Department of Medicine, University of Oxford, Oxford, OX3 7FZ UK; 5 School of Nursing & Midwifery, University of Technology, Sydney, Australia; 6 PRAXIS Forum, Green Templeton College, University of Oxford, Oxford, OX2 6HG UK; 7 School of Medicine, Griffith University, Queensland, Australia

**Keywords:** Nurse, Nursing, Nurse-sensitive indicators, Metrics, Quality nursing care and outcome measures

## Abstract

**Background:**

The use of appropriate and relevant nurse-sensitive indicators provides an opportunity to demonstrate the unique contributions of nurses to patient outcomes. The aim of this work was to develop relevant metrics to assess the quality of nursing care in low- and middle-income countries (LMICs) where they are scarce.

**Main body:**

We conducted a scoping review using EMBASE, CINAHL and MEDLINE databases of studies published in English focused on quality nursing care and with identified measurement methods. Indicators identified were reviewed by a diverse panel of nursing stakeholders in Kenya to develop a contextually appropriate set of nurse-sensitive indicators for Kenyan hospitals specific to the five major inpatient disciplines. We extracted data on study characteristics, nursing indicators reported, location and the tools used. A total of 23 articles quantifying the quality of nursing care services met the inclusion criteria. All studies identified were from high-income countries. Pooled together, 159 indicators were reported in the reviewed studies with 25 identified as the most commonly reported. Through the stakeholder consultative process, 52 nurse-sensitive indicators were recommended for Kenyan hospitals.

**Conclusions:**

Although nurse-sensitive indicators are increasingly used in high-income countries to improve quality of care, there is a wide heterogeneity in the way indicators are defined and interpreted. Whilst some indicators were regarded as useful by a Kenyan expert panel, contextual differences prompted them to recommend additional new indicators to improve the evaluations of nursing care provision in Kenyan hospitals and potentially similar LMIC settings. Taken forward through implementation, refinement and adaptation, the proposed indicators could be more standardised and may provide a common base to establish national or regional professional learning networks with the common goal of achieving high-quality care through quality improvement and learning.

## Background

Globally, there is a growing concern about the need for quality health care, with a view that poor-quality care provision is not only wasteful but also ineffective and unethical [1]. Measurement of quality indicators is central to improvement efforts aimed to promote accountability in healthcare and professional practice. Quality indicators arise from the increasing demand for measures of quality across the healthcare continuum ranging from the community to tertiary level [2]. Nurses form the largest component of the health professional workforce and are recognised as essential to the delivery of safe and effective care. Understanding, measuring and reporting the quality of their work is, therefore, critical.

Quality assurance in nursing requires that nurses have the ability to measure their care, to define standards and to change their professional practice [3]. Therefore, measuring what nurses do is important in maintaining standards, supporting nursing management and understanding outcomes and their variation that is linked to nursing. This requires development of sensitive, nursing-specific indicators [4]. Nurse-sensitive indicators (NSIs) have been identified and used by healthcare organisations and researchers to measure how much nurses contribute to patient outcomes [5, 6]. Although there are varied definitions of NSIs, the most comprehensive one defines NSIs as measures of things that are about nursing (structure), about what nurses do (process) or about outcomes that can be linked to structure and process issues. These measures must be quantifiably influenced by nursing personnel, but the relationship between these measures and nursing is not necessarily causal [7].

The use of appropriate and relevant key performance indicators for nursing provides an opportunity to (i) demonstrate the unique contribution nurses make in delivering outcomes for patients and clients [8], (ii) highlight the gaps that might exist in nursing care provision, (iii) inform intervention design for improving nursing care provision and (iv) promote accountability for the care that nurses provide. With a focus on the inpatient setting and the potential use of NSIs for evaluating and improving quality in low- and middle-income countries, our aims were to (i) use a scoping review to identify NSIs reported in the literature and (ii) through a stakeholder-led approach, to adapt and if needed expand NSIs for potential use in Kenyan hospitals, and (iii) to develop a set of indicators with the potential use in wider LMIC contexts to support future evaluations of nursing care provision.

## Methods

### Review of literature

A scoping review [9, 10] undertaken to identify the literature on metrics for nursing quality of care, nursing care quality and their measurement methods (tools and data collection approaches) was conducted using EMBASE, CINAHL, MEDLINE and Google Scholar databases. The literature search was conducted using the following search terms: nurs* care metrics, nurs* care indicators, nurs* services indicators, nurs* metrics, nurs* care measures, and quality of care or nursing care.

### Study selection criteria

We searched for all relevant literature published in the English language (due to time constraints) between 1900 and April 2017. Bibliographic references of retrieved studies were searched for additional articles that reported nursing quality indicators or nursing metrics. All study designs from all settings (LMIC, and high-income countries (HIC)) which reported on nursing care services and had an explanation of the concept of the quality of nursing care, and their measurement methods were included. Studies that reported ambulatory nursing care were excluded since the focus of the study was to develop indicators for the inpatient setting.

**Fig. 1:**
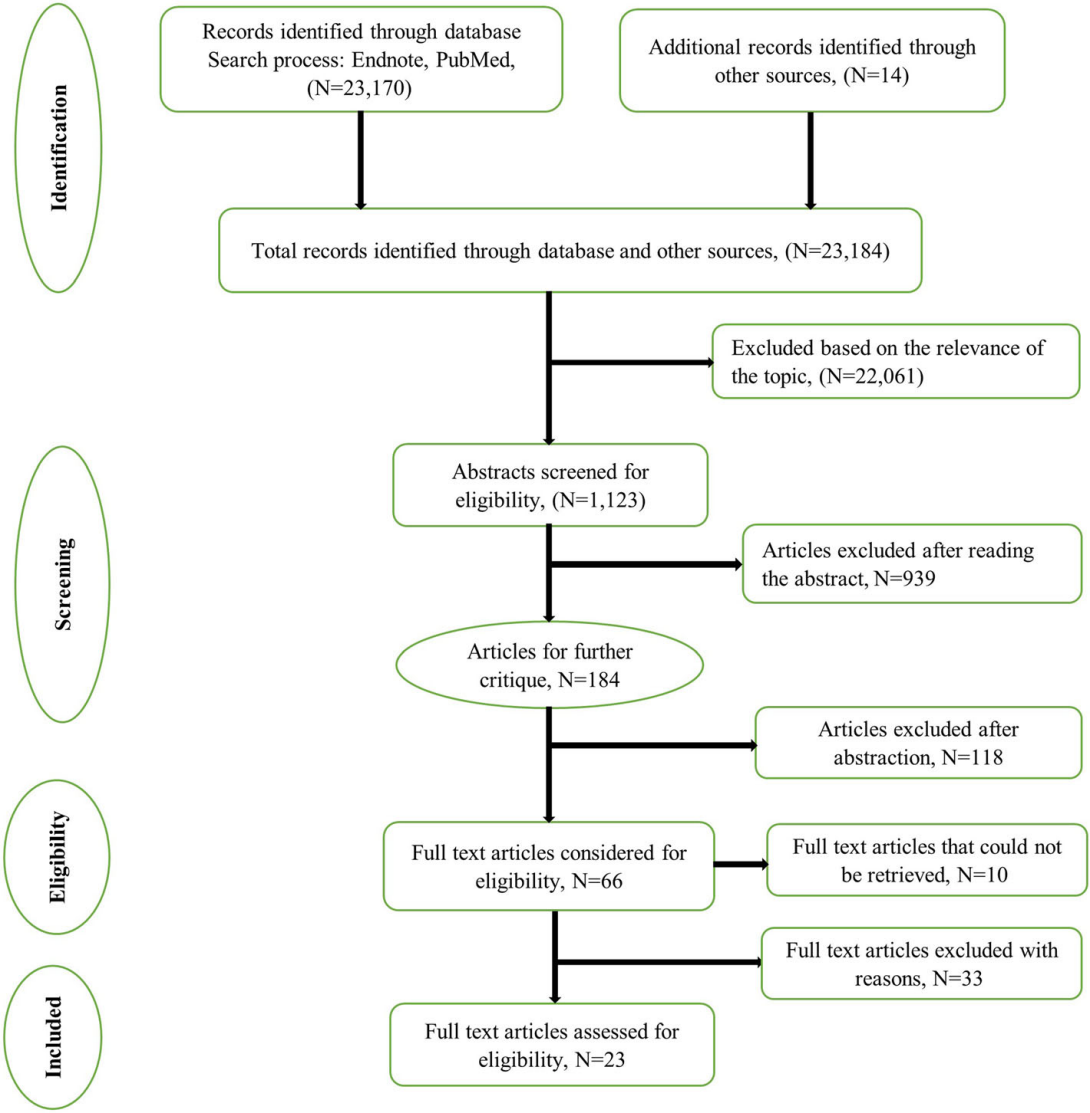
PRISMA flow chart on the literature search process. The PRISMA flow diagram for the selection process of studies and reasons for exclusion

All titles and abstracts of identified articles were screened by two reviewers (DG and MZ) independently, and any disagreements resolved by discussion. Full texts of potentially relevant papers were retrieved, read and subjected to the inclusion/exclusion criteria. The authors did not assess the quality of the selected studies as our interest was in capturing a full list of indicators rather than how or how well they have been used. The process and reporting, including the step-wise retrieval, review, appraisal and inclusion into the study of literature (Fig. 1), followed the preferred reporting items for scoping reviews as outlined in the PRISMA extension for scoping reviews statement [11].

### Data extraction and synthesis

Data on study characteristics (e.g. study design, settings, objectives, sample size, discipline/unit), nursing indicators reported, study location and tools used (including availability) were abstracted on a standardised form and are summarised in Additional file 1. The abstraction was completed by one reviewer (MZ); a second reviewer (DG) counter-checked the extracted data. The primary reviewers (DG and MZ) discussed and resolved any differences in perspective that arose during the review to arrive at the final studies for inclusion. Agreement was achieved by consensus.

All of the identified publications mentioned indicators (159) and the studies which included them (across the 23 studies identified) were listed. The data requirements for these indicators were also explored in terms of data source and how to calculate the indicator (numerator/denominator). The indicators were then categorised narratively into three broad overlapping themes (allowing indicators to be in one or more categories) to inform the stakeholder-led process for selection of potential indicators applicable to Kenyan hospitals. The three broad thematic areas identified were (i) commonly reported indicators (identical indicators in four or more studies), (ii) indicators characterised into the respective domains of the Donabedian quality of care model (structure, process and outcome) and (iii) in the opinion of the authors (DG and MZ are both nurses and familiar with the public hospital settings in Kenya), indicators relevant and with potential direct application to Kenya with minor modifications. Indicators reported in the literature linked to other classifications/domains of quality (for instance compassion, safety or patient perspective) were re-categorised into the Donabedian framework based on the authors’ judgement on what domain the indicator best represented.

### Stakeholder engagement to adopt/adapt indicators for the Kenyan context

To develop and contextualise a set of NSI to support evaluations of nursing care provision in Kenyan hospitals and wider LMIC settings, we established an expert advisory group (described below) to provide recommendations on what indicators would be contextually appropriate to measure nursing care in an LMIC setting. We presented findings from the scoping review and used the National Quality Forum (NQF) framework [12] on developing indicators for public reporting to guide the advisory panel on the selection of indicators from those identified in the review or develop new ones where necessary. NQF is a consensus-based health care organisation in the United States of America that defines measures or health practices that are the best, evidence-based approaches to improving care [13].

#### Selection of stakeholders

Drawing on our prior work with a broad neonatal stakeholder group [14, 15], we established an expert advisory group comprising individuals responsible for delivery of nursing care in major public hospitals, neonatal nurse training and nursing services policy in the Ministry of Health and County Governments. We also included major nursing stakeholder groups including the National Nurses Association of Kenya, the Nursing Council of Kenya and development partners (WHO, UNICEF).

The nursing advisory group was aimed at gaining a broad representation of the nursing community rather than a statistically representative group. We constituted panels from the nursing advisory group which met on two occasions for a full day of consultations. In the first meeting, a high-level group (*n* = 26) involved in policy-making drawn from the nursing directorate at the national level, training and regulatory institutions, and development partners met to review indicators identified through the scoping review with discussions being focused on a pre-identified list of possibly relevant indicators for LMIC selected by the authors. After a plenary session, smaller groups of at least five members, organised so that each group had broad representation in expertise and institutional affiliation, were formed to discuss indicators relevant to inpatient care for the five major inpatient disciplines (surgery, medicine, paediatrics, neonatal care and obstetrics and gynaecology). These discipline-specific groups were tasked with recommending a list of indicators for use in Kenya for the respective disciplines based on the literature in the form of the author’s pre-identified list. On average, each group reviewed 10–15 of the pre-identified indicators. Additionally, group members were allowed to propose new indicators that were not captured in the literature but were deemed appropriate for the Kenyan context based on their experience and expertise which would then be considered by the entire panel. The discussions on indicator selection and prioritisation drew on the guidance from the National Quality Framework (NQF) [12] and focussed on (i) which indicators were relevant and important to these disciplines in representing the quality of nursing care, (ii) acceptability by the nursing profession that the indicator was an important aspect of their work and that its measurement would be a credible as an assessment of their work, (iii) availability of existing data sources that could support evaluations and (iv) where data were not routinely available, whether it would be feasible/realistic to introduce new data elements. After deliberations, each of the discipline-specific groups presented their propositions to the wider advisory group, and consensus on what indicators should finally be proposed was sought through discussion and show of hands.

In the second meeting, the final list of indicators proposed from the initial high-level stakeholder group was presented to a group of 10 front line nurses (two nurses practising in each of the disciplines) for further refinement and prioritisation. This group was not mandated to reject indicators but advised on how to measure these indicators in practice.

The final list of indicators arising from the stakeholder-led process was categorised against the International Patient Safety Goals (IPSG) domains [16] and the Donabedian framework in instances where no suitable domain on the IPSG criteria was identified. The IPSG criteria were developed by the Joint Commission International (JCI) which is a recognised leader in international health care accreditation and focuses on identifying, measuring and sharing best practices in quality and patient safety [17].

## Results

### Overview of the studies included in this review

Overall, we identified 23 170 articles from database searches and an additional 14 articles from reference lists and Google Scholar. After screening titles and abstracts, 66 articles were considered for full-text review; however, 10 articles were not reviewed because full-text articles were inaccessible to us (*n* = 6) or they were not available in English (*n* = 4). Of the 56 full-text articles retrieved, 23 articles met our inclusion criteria. The main reasons for exclusion were that articles reported on ambulatory care indicators, described the process of developing and testing NSIs or were descriptions of how the NQF endorsed indicators might be implemented in practice and their potential impact. The article selection process is presented in the PRISMA flow chart (Fig. 1).

The reviewed studies included ten that collected primary data, two systematic reviews, three reports, one expert opinion and seven narrative reviews. A detailed description of the studies reviewed is provided in Additional file 1. The primary studies focussed on different settings such as specialist units (inpatient cardiovascular and critical care units, *n* = 3) and more general settings (acute care settings, medical/surgical units, swing bed units and transitional care, *n* = 7). The countries in which studies were conducted varied, most (*n* = 10) were conducted in the United States of America, followed by Europe (*n* = 6), Asia (*n* = 5) and Australia (*n* = 2). Within a single study, the minimum number of indicators was 6, the maximum was 44 and the median was 11 (IQR 7–17). Study type, setting, number of indicators reported and country where the study was done are reported in Table 1.

**Table 1:** The characteristics of the studies included in this review

Author	Title	Sample size	Aim and setting	Study method	Indicator domain (number)	Study location
Kunaviktikul et al. 2005	Development of indicators to assess the quality of nursing care in Thailand	Not specified	General clinical nursing	Descriptive observational (FGDs observation sheets, record retrieval forms)	9 indicators; Structure (2), process (2), outcome (5)	Thailand (Asia)
McCance et al. 2012	Identifying key performance indicators for nursing and midwifery care using a consensus approach	130	General nursing and midwifery	Consensus (collaborative problem solving) method	6 process indicators	Ireland (Europe)
Langemo et al. 2002	Nursing quality outcome indicators: The North Dakota Study	217 nurses; 924 patients	Medical and surgical units, intensive care units, transitional care, and swing bed units	Expert/questionnaire	11 indicators: structure (3), process (3), outcome (5)	North Dakota (United States of America)
Pazargadi et al. 2008	Proposing indicators for the development of nursing care quality in Iran	161 nurses	General clinical nursing	Descriptive-exploratory	20 indicators: structure (10), process (5), outcome (5)	Iran (Asia)
La Sala et al. 2017	The quality of nursing in intensive care: a development of a rating scale	43 experts	Intensive care unit	Literature review and panel of experts	21 process indicators.	Italy (Europe)
Fugaça et al. 2015	Use of balanced indicators as a management tool in nursing	200 medical records	Intensive care unit	Case study	14 indicators: structure (1), process (7), outcome (6)	Brazil (United States of America)
Burston et al. 2013	Nurse-sensitive indicators suitable to reflect nursing care quality: a review and discussion of issues	40 studies	General nursing	Review	44 outcome indicators	Australia
Foulkes et al. 2011	Nursing metrics: measuring quality in patient care	Not specified	General nursing	Expert opinion	10 indicators: safety (5), effectiveness (3), nurses compassion (2)	United Kingdom (Europe)
Chen et al. 2016	Using the Delphi method to develop nurse-sensitive quality indicators for the NICU	41 experts	Neonatal intensive care units	Modified Delphi technique	11indicators: structural (1), process (2), outcomes (8)	China
Seaman et al. 2016	Abstracting ICU nursing care quality data from the electronic health Record	1 440 case records	Intensive care unit	Single-blind, randomised crossover cluster (stepped wage) design	6 indicators	Pennsylvania (United States of America)
Martha et al. 2006	The nightingale metrics	Not specified	General nursing	Focused group discussion	Inpatient cardiology unit (4), PICU (7), CICU (6), NICU (8)	Boston (United States of America)
Twigg et al. 2015	Foundation of nurse-sensitive outcome indicator suite for monitoring public patient safety in Western Australia	259 463 patient records	Medical and surgical units	A review of literature and piloting of indicators on an EHR	8 outcome indicators	Australia
Maben et al. 2012	High quality metrics for nursing.	18 experts	General nursing	Taskforce review	34 indicators: safety (9), effectiveness (5), patient experience (10), workforce (5), staff experience (5)	United Kingdom (Europe)
Griffiths et al. 2008	State of the art metrics for nursing: a rapid appraisal	Not applicable	General nursing	Review	18 indicators: safety (7), effectiveness (8), compassion (3)	United Kingdom (Europe)
Koy et al. 2016	The quantitative measurement of nursing care quality: a systematic review of available instruments	18 tools	General nursing	Systematic review	Nurses’ perspectives (11), patients’ perspectives (5). Categories and subcategories of nurse-patient perspectives	Cambodia (Asia)
McCance et al. 2009	Using the caring dimensions inventory as an indicator of person-centred nursing.	107 patients; 122 nurses	Medical and surgical, ICU, operating room, sexual health clinic, older people rehabilitation and paediatric infectious disease wards	Quasi-experimental	40 indicators: nurses’ perspectives (19), patients’ perspective (21) Both nurses and patients (6)	United Kingdom (Europe)
Montalvo et al. 2007	The national database of nursing quality indicators		General nursing	Report	14 indicators: structural (4), process (1), outcome (4), outcome/process (4)	United States of America
Zhang et al. 2016	Assessing nursing quality in paediatric intensive care units; a cross sectional study in China.	1 385 patients and 274 PICU nurses.	Paediatric intensive care units	Descriptive, cross-sectional	15 indicators: structural (5), process (3), outcome (7)	China
Riehle et al. 2007	Specifying and standardizing performance measures for use at a national level; implications for nurse-sensitive care performance measures.		General nursing	Report	35 outcome indicators	United States of America
Lacey et al. 2006	Developing measures of paediatric nursing quality	10 acute care hospitals	Paediatric units	Review of literature, panel of experts and pilot study	6 outcome indicators	United States of America
Stratton et al. 2008	Paediatric	34 patient care units.	Paediatric units	Descriptive, Correlational, linear mixed model.	9 indicators	United States of America
St Pierre et al. 2006	Staff nurses’ use of report card data for quality improvement		General nursing	Report	14 indicators	United States of America
Lacey et al. 2009	Nursing; key to quality improvement		General nursing	Review	15 indicators: patients centred (8), nursing-centred (3), system-centred (4)	United States of America

*CICU* cardiac intensive care unit, *FGD* focused group discussion, *HER* health electronic records, *KPI* key performance indicator, *NHS* national health service, *NICU* neonatal intensive care unit, *PICU* paediatric intensive care unit, *SOP* standard operating procedure

Different authors had different approaches for classifying nurse-sensitive indicators. In a study conducted by Foulkes aiming at enhancing the understanding of nursing metrics in clinical practice in the United Kingdom, nursing indicators were categorised into safety, effectiveness and compassion in nursing care [18]. The High-Quality Care Metrics for Nursing report categorised the quality outcome into safety, effectiveness and experience of the care provision (both nurses and patients) categories [19]. In the review by Koy and colleagues, indicators were classified into nurse perspectives, patient perspectives and nurse-patient perspectives based on who’s perception of quality the indicator was measuring [20]. McCance et al. also reported patient and nurse perceptions of caring based on the patient-centred nursing framework [8]. The most commonly adopted approach by authors was the empirical framework for quality of care assessment of health systems by Donabedian that focuses on the structure, process and outcome domains [21]. There were variations in the domains reported with studies reporting indicators in all three [22–24] or one of three domains without explicitly mentioning which domain these indicators belonged to [6, 25–27]. A summary of the indicators reviewed and the domain they were categorised into as per the Donabedian quality care model is presented in Table 2.

**Table 2:** Nurse-sensitive indicators identified from the literature and classified as per the Donabedian quality framework (indicators have been extracted as reported in the literature, and indicators with similar definitions or measuring the same construct are included)

Outcome	
Failure to rescue	Pain presence
Postoperative respiratory failure	Patient satisfaction with pain management
Patient complaints	Pain management/controlled
Patient satisfaction with educational information	Nurse staff satisfaction
Patient satisfaction with nursing care	Physical well-being
Patient satisfaction with overall care	Psychological wellbeing
Patients’ confidence in knowledge and skills of the nurse	Iatrogenic lung collapse
Patient’s sense of safety whilst under the care of the nurse	Atelectasis
^a^Patient involvement in decisions about their nursing care	Fluid overload
Respect from the nurse for patient’s preference and choice	Falls
Nurse’s support to patients to care for themselves, where appropriate	Injuries to patient
Nurse understanding of what is important to the patient	Patient’s falls with injuries in the hospital
Patient satisfaction with nurse communication	Staff injuries on the job
Patients experience of care	Knowledge, behaviour, status change scores
Patient/family complaints satisfaction	Physical and mental health change scores
Parent/family complaint rate	Follow-up rate to allergy risks
Patient judgement of hospital quality	Adverse drug reaction rate
Central line catheter-associated bloodstream infection	Total of prescription mistakes
Hospital-acquired pneumonia	Total of transfusion reaction
Respiratory tract infection	Upper GI bleeding
Nosocomial infection	Mortality
Ventilator-associated pneumonia (VAP)	Shock/cardiac arrest
Wounds dressed	Deep vein thrombosis
Intravenous/vascular access infection	CNS complications
Thrombophlebitis	Deterioration
Vascular access infiltration	Complications
Vascular access thrombosis	Health status
Peripheral venous extravasation	Symptom management index
Hospital-acquired urinary tract infection	Symptom resolution
Urinary catheter-associated UTI	Metabolic derangement
Wound infection	Functional status
Surgical wound infection	Rate of accidental endotracheal extubation
Sepsis	Retinopathy of the preterm child (ROP)
Intravascular infiltration due to IV therapy	Heavy sedation
Gastrointestinal infection rate	Average hospital length of stay
Pressure ulcer prevalence	Vaccination
Psychiatric physical/sexual assault rate	
**Process**	
Wound care	Smoking cessation counselling
Skin integrity/pressure ulcer prevention	Smoking cessation counselling for heart failure
Decubitus prevention care	Smoking cessation counselling for pneumonia
The risk factors for pressure sores have been documented	Smoking cessation counselling for acute myocardial infarction (AMI)
Pain assessment with scale and recorded	Nursing care supervision
Chest-abdomen drain changed as by the protocol	Assessment and record reflex presence (e.g. ocular)
Chest-abdomen drain insertion area dressed as by guideline	Proper patient positioning in bed
Mechanical ventilation has been replaced according to protocols	Monitor alarms properly set
Body temperature values have been updated in the last 24 h	ABG result 1 hour after endotracheal tube removal is available
The pulse oximetry has been monitored and recorded	Endotracheal suctioning performed as per prescription and recorded
The ECG and vital signs have been recorded on admission	Hand washing and hand hygiene
Measuring of patient observations (vital signs)	Documentation of results
Fluid intake and output have recorded	Number of patient transfers
Patient washing once a day and recorded	Double-checking of all medication by two nurses
Patient mouth washing as by ward procedure and recorded	Weight documentation daily
^a^Assisting a patient with activities of daily living	Relative/parent notification of patients transfer
^a^Instructing patient about self-care	Unplanned admission
^a^Being honest with a patient	Interprofessional relations
^a^Keeping relatives informed about a patient	Emergency care
^a^Providing privacy for a patient	Discharge and case management
^a^Getting to know the patient as a person	Appraisal and induction
^a^Giving reassurances about a clinical procedure	Nurses’ compliance in filling of medical records
Information and involvement of family into the end of life care by nurses	^a^Listening to a patient
Physical and chemical restraint	^a^Explaining a clinical procedure to a patient
^a^Medication errors	^a^Being with a patient during a clinical procedure
Antithrombotic therapy given and recorded at the correct time	^a^Consulting with a doctor
^a^Reporting a patient’s condition to a senior nurse	^a^Observing the effects of medication on a patient
**Structure**	
Satisfaction questionnaire about work periodically administered to nurses	level of education and work experiences of nurse managers
Total nursing care hours provided per patient day	Nursing continuing education
Skill mix (mix of RNs, LPNS and unlicensed staff)	In-service education hours for nursing staff per year
Number of nurses per patient	Educational materials for nurses in the hospital (library, internet, etc.)
Working hours of nursing staff	Organisational goal setting
Proportion of nurses working more than 3 years (nurses experience)	Nursing job description
Nurse bed care ratio	Organisational budgeting for patient safety
Voluntary nurse staff turnover rates	Patient waiting time for nursing care
Patient to nurse ratio	Nursing care standards in hospitals
Nurse vacancy rate	Safety environment for nurses in hospital
Overtime	Practice environment scale-Nursing Work Index
Understaffing as compared to the organisation’s plan	Noise
On-call or per diem use	Emergency equipment/drugs available
Sick time	Total volume of laundry per patient
Agency staff use	Visitation policy
Staffing level of education	Absenteeism

^a^Indicators used to measure nursing quality from a nurse or patient perspective

### Indicators relevant for LMICS

Of the 159 indicators identified from the literature, the authors identified 70 indicators relevant to LMIC settings based on their understanding and experience in this context. These were then presented to the stakeholder group for consideration for use in LMIC hospitals. Of these, 31 indicators were adopted by stakeholders through the consensus process. These indicators were revised and clarified to take into account the Kenyan context. An additional 34 indicators were proposed by the stakeholder group based on the need and priority to monitor specific aspects of nursing care in LMIC. Of these, 21 indicators were adopted after deliberation and based on panel consensus. In total, 52 NSIs potentially relevant to LMIC settings were identified. This included 14 of the 25 commonly reported indicators (reported in at least four or more studies) presented in Additional file 2. A detailed description of the indicators adapted from existing indicators (literature), those recommended as additional indicators and the proposed methods for measuring the indicators as suggested by the stakeholder group is provided in Table 3.

**Table 3:** LMIC relevant Nursing sensitive indicators aligned with International Patient Safety Goals

International patient safety goals domain	Indicator definition	Source of indicator	Measurement approach
Identify patients correctly			
	Proportion of patients with name tags	Literature (IPSG)	Structure
Improve effective communication			
	Proportion of patients who have a complete assessment (history, head to toe examination, vital signs, weight/height, plan of care) at admission	Literature	Process
	Proportion of patients who have discharge instructions (follow-up care, education, return date)	Literature	Process
	Proportion of patients with appropriate vital signs monitoring as per patient acuity documented	Literature	Process
	Proportion of patients who received at least one session of counselling or communication in 24 hours	Literature	Process
	Proportion of patients with assessment and planning of care done at least once in 24hours	Literature	Process
	Proportion of patients with ward round recommendations documented in the cardex	Stakeholders	Process
	Proportion of patients with surgeons’ instructions transferred to the cardex and with completely filled postoperative forms	Stakeholders	Process
	Availability of basic nursing forms/charts	Stakeholders (HFA)	Structure
	Adverse effects reporting system in place to reporting	Stakeholders (HFA)	Structure
Improve the safety of high-alert medications			
	Record of daily stock monitoring/handover and safety of drugs classified under the Dangerous Drugs Act	Stakeholders	Structure
	Proportion of blood transfusions monitored as per blood transfusion guidelines	Literature	Process
	Proportion of documented blood transfusions reactions	Literature	Outcome
	Proportion of patients on anti-coagulation therapy with dose, drug and food interactions, and appropriate nursing care documented	Literature (NPSG)	Process
	Proportion of patients on drugs with a narrow therapeutic range that are flagged	Literature (NPSG)	Process
Ensure correct site, procedure, patient for surgery			
	Proportion of patients scheduled for surgery with correctly and completely filled preoperative forms/checklist	Stakeholders	Process
	Proportion of patients with the status of the patient, surgical procedure and surgical site, documented in the cardex	Literature (IPSG)	Process
	Proportion of patients with filed consent form before surgery	Stakeholders	Process
	Proportion of patient identifiers before surgery (name tags/other identifying measures)	Literature (IPSG)	Process
	Proportion of patients with pre-marked sites for procedures that require marking of the incision or insertion site.	Literature (IPSG)	Process
Reduce risk of HCA infections			
	Proportion of surgical patients with post-operative surgical wound infection	Literature	Outcome
	Proportion of patients on intravenous fluids/treatment whose cannula site was checked and documented (state of cannula site- swollen, SSI, soiled)	Literature	Outcome
	Proportion of patients on intravenous fluids/treatment whose cannula site was checked and documented vascular access infiltration	Literature	Outcome
	Proportion of patients requiring wound cleaning with wound cleaned and wound dehiscence (wound characterization-burst wound, septic, granulating, necrotic), exudate and pain documented	Literature	Process
	Proportion of newborns aged <5 days and born within the hospital who develop septic cords	Stakeholders	Outcome
	Proportion of newborns on phototherapy with documentation of eyecare done, eyes checked for damages and eye pad changed once in 24 hours	Stakeholders	Process
	Proportion of patients with UTI in non-genito urinary infection with documentation for input-output monitoring	Literature	Outcome
	Proportion of patients who develop pressure ulcers while in the ward	Literature	Outcome
	Proportion of patients with basic activities of daily living (ADL) done.	Literature	Process
	Compliance with hand hygiene guidelines based on established goals	Literature	Process
	Patient education on infection prevention practices	Stakeholders	Process
	Availability of hand hygiene guidelines/training/reminders	Stakeholders (HFA)	Structure
	Availability and easily accessible clean toilets	Stakeholders	Structure
	Availability of Waste segregation (3 bins and sharp boxes)	Stakeholders (HFA)	Structure
	Needle, sharp box more than 3/4 full, or any used needles/sharps outside the box	Stakeholders (HFA)	Structure
	Bandages/infectious waste lying uncovered	Stakeholders (HFA)	Structure
	Clean running water (piped, bucket with tap, or pour pitcher)	Stakeholders (HFA)	Structure
	Functioning hand hygiene stations (that is, alcohol-based hand rub solution or soap and water with a basin/pan and clean single-use towels)	Stakeholders (HFA)	Structure
	Storage space for sterile and high-level disinfected items (either a room with limited access or a cabinet that can be closed)	Stakeholders (HFA)	Structure
Reduce risk of patient harms resulting from falls			
	Proportion of patients with risk of falling who have harm reduction measures	Literature	Process
	Use of physical restraint	Literature	Process
	Proportion of patient falls with injuries	Literature	Process
Additional indicators that don’t fall in the IPSG criteria			
Other safety related indicator			
	Proportion of patients at risk of DVT (immobile, obese, on total nursing care etc) who are assessed for DVT at least once in 24 hours	Literature	Process
	Proportion of diabetic and critically patients with blood sugar monitoring	Stakeholders	Process
	Proportion of diabetic patients with the following documented: type of feed, medication, frequency, intervention, sugar levels, time of last feed to help interpret the result)	Stakeholders	Process
Structure indicators			
	Patient to nurse ratio	Literature	Structure
	Nurse skill mix (by education level)	Literature	Structure
	Staff wearing name tags and on uniform	Stakeholders (HFA)	Structure
Outcome indicators			
	Patient satisfaction with overall care	Literature	Outcome
	Patient satisfaction with nursing care	Literature	Outcome
	Proportion of patients who died	Literature	Outcome
	Average length of stay (by illness acute vs chronic)	Literature	Outcome

**Literature** - Indicator identified from the systematic adopted for LMIC/Kenyan context; **Stakeholder** - Indicator not defined in literature but stakeholders felt this was a priority/important area to measure; **IPSG/NPSG** - Indicator has been defined under either of these criteria; **Stakeholder (HFA)** - indicator already exists in the Joint Health Facility Assessment (HFA) indicator set developed through a stakeholder process

*UTI* Urinary tract infection, *DVT* Deep venous thrombosis, *HCA* Health care acquired

## Discussion

The aim of this study was to identify from the literature ‘nurse-sensitive indicators’ (NSIs) and, use a stakeholder-led approach, to develop and contextualise potential indicators to support evaluations of nursing care provision in Kenyan hospitals and potentially similar LMIC settings. Although there were several studies reporting NSIs, there were inconsistencies in the terminologies/definitions used to describe nursing quality indicators including nurse-sensitive indicators, nursing key performance indicators, nurse-sensitive quality indicators and nursing metrics [2, 5, 6, 20, 28]. In addition, definitions used for indicators varied by tool and data source despite the indicators aiming at assessing the same practice or outcome. For instance, nosocomial infections are considered in the aggregate in some studies whilst others described them by the system affected or resulting diseases such as urinary tract infections, pneumonia and upper respiratory infections. For example, some studies reported pneumonia and ventilator-acquired pneumonia as separate indicators (Table 4) [6, 29]. Consequently, there is considerable overlap in measurement approaches and limited standardisation across indicators undermining comparison between organisations or hospitals. Given the costs of measurement and the limited resources in LMICs, it will be important that a consistent and standard approach to indicator definition and measurement is developed to support the evaluation of nursing care in these settings.

**Table 4:** Indicators with similar definitions or measuring similar construct

Broad indicator definition	Indicators as defined in the literature
Failure to rescue	Failure to rescue
	Postoperative respiratory failure
Patient satisfaction	Patient complaints
	Patient satisfaction with educational information
	Patient satisfaction with nursing care
	Patient satisfaction with overall care
	Patients’ confidence in knowledge and skills of the nurse
	Patient’s sense of safety whilst under the care of the nurse
	Patient involvement in decisions made about their nursing care
	Respect from the nurse for patient’s preference and choice
	Nurse’s support to patients to care for themselves, where appropriate
	Nurse understanding of what is important to the patient
	Patient satisfaction with nurse communication
	Patients experience of care
	Patient/family complaints satisfaction
	Parent/family complaint rate
	Patient judgement of hospital quality
Hospital-acquired infection	Central line catheter-associated bloodstream infection (CLABSI)
	Hospital-acquired pneumonia
	Respiratory tract infection
	Nosocomial infection
	Ventilator-associated pneumonia (VAP)
	Wounds dressed
	Intravenous/vascular access infection
	Thrombophlebitis
	Vascular access infiltration
	Vascular access thrombosis
	Peripheral venous extravasation
	Hospital-acquired urinary tract infection
	Urinary catheter-associated UTI
	Wound infection
	Surgical wound infection
	Sepsis
	Intravascular infiltration due to IV therapy
	Gastrointestinal infection rate
	Wound care
Pressure ulcer	Pressure ulcer prevalence
	Skin integrity/pressure ulcer prevention
	Decubitus prevention care
	The risk factors for pressure sores have been documented
Pain management	Pain presence
	Patient satisfaction with pain management
	Pain management/controlled
	Pain assessment with scale and recorded
Job satisfaction and health worker well-being	Nurse staff satisfaction
	Physical well-being
	Psychological wellbeing
	Satisfaction questionnaire about work periodically administered to nurses
Staffing and skill mix	Total nursing care hours provided per patient day
	Skill mix (mix of RNs, LPNS and unlicensed staff)
	Number of nurses per patient
	Working hours of nursing staff
	Proportion of nurses working more than 3 years (nurses experience)
	Nurse bed care ratio
	Voluntary nurse staff turnover rates
	Patient to nurse ratio
	Nurse vacancy rate
	Overtime
	Understaffing as compared to organisation’s plan
	On-call or per diem use
	Sick time
	Agency staff use
	Staffing level of education
	Level of education and work experiences of nurse managers
	Absenteeism
Nursing education	Nursing continuing education
	In-service education hours for nursing staff per year
	Educational materials for nurses in the hospital (library, internet, etc.)
Respiratory support or failure	Iatrogenic lung collapse
	Atelectasis
	Chest-abdomen drain changed as by the protocol
	Chest-abdomen drain insertion area dressed as by guideline
	Mechanical ventilation has been replaced according to protocols
Vital signs monitoring	Body temperature values have been updated in the last 24 h
	The pulse oximetry has been monitored and recorded
	The ECG and vital signs have been recorded on admission
	Measuring of patient observations (vital signs)
Fluid input output monitoring	Fluid overload
	Fluid intake and output have recorded
Activities of daily living	Patient washing once a day and recorded
	Patient mouth washing as by ward procedure and recorded
	Assisting a patient with activities of daily living
	Self-care
Nursing support and communication to patients	Being honest with a patient
	Keeping relatives informed about a patient
	Providing privacy for a patient
	Getting to know the patient as a person
	Giving reassurances about a clinical procedure
	Information and involvement of family into the end of life care by nurses
Falls	Falls
	Injuries to patient
	Patient’s falls with injuries in the hospital
	Staff injuries on the job
	Physical and chemical restraint
Medical/nursing errors	Adverse drug reaction rate
	Total of prescription mistakes
	Total of transfusion reaction
	Medication errors
Counselling	Smoking cessation counselling
	Smoking cessation counselling for heart failure
	Smoking cessation counselling for pneumonia
	Smoking cessation counselling for acute myocardial infarction (AMI)

Using a stakeholder-driven approach, indicators identified from the literature were reviewed for relevance to a LMIC setting and where necessary initially adapted by discipline-specific groups (surgery, medicine, paediatrics, neonatal care and obstetrics and gynaecology). Of the 159 indicators identified, 70 were considered by researchers familiar with the local context and with quality measurement as potentially relevant to LMIC hospital settings. Of these, 31 were selected (and often adapted) by local stakeholders as likely to be useful for the Kenyan context. The reasons why indicators were excluded spanned different case-mix of patients and hospital settings including the availability of technology and infrastructure in HICs that were often lacking in LMICs. An additional 21 indicators that were not identified in the literature were recommended by stakeholders to measure aspects of nursing care provision that were considered a priority for the Kenyan context. These additional indicators spanned the domains of structure assessment (e.g. availability of resources to support infection prevention and control activities/practices) and process (e.g. monitoring of phototherapy, communication and coordination of care through documented doctor’s ward rounds and consenting for surgical procedures). Our final set of indicators (*n* = 52) was classified based on the International Patient Safety Goals (IPSG) framework [16] (Table 3) and spanned all the domains of patient identification (*n* = 1); effective communication (*n* = 9); safety of high-alert medication (*n* = 5); correct site, procedure and patient for surgery (*n* = 5); risk of health care-acquired infections (*n* = 19); and patient harms resulting from falls (*n* = 3). Developing measurements of the work done by nurses and a link to patient safety may be important in helping us understand the consequences of workforce shortages, and such measures could be helpful in accreditation programmes emerging in LMICs [30–32] whilst drawing lessons from global programmes such as the Joint Commission International (JCI) [33].

Progress has been made in defining, refining and testing NSIs in HICs with the development of nursing networks that use NSIs for quality improvement. Examples of these include the adoption and widespread use of the American Nurses Association National Database of Nursing Quality Indicators (NDNQI) in evaluating the nursing quality of care [34] and the creation of minimum datasets for nursing quality indicators [35], but all these are limited to HICs. Exploring the commonly reported NSIs in HIC settings for transferability to LMIC with the premise that these are the most robust indicators based on their prevalent use, only 14 out of the 25 commonly reported indicators were adopted by stakeholders (Additional file 2). This suggests varying contexts and needs that should be considered when adapting recommendations from other settings. Therefore, approaches and progress made provide important lessons for LMICs as they consider indicators for adoption and operationalisation to avoid pitfalls that might have been experienced by HICs during the processes of setting up these systems. We hope by developing NSIs for a LMIC setting and using lessons on their implementation from HIC will help demonstrate the value, importance and broader contribution of nursing to high quality care both at local and wider levels whilst exploring what might constitute a minimum data set that allows quality monitoring and risk adjustment.

Nurses, the largest component of the health professional workforce, are essential to the delivery of safe and effective care as there are very few interventions (both clinical and nurse initiated) that occur without nursing involvement. Whilst nurses comprise the largest workforce and are considered the ‘glue’ that holds the health care system together, they are too often undervalued and their contribution to the quality of care agenda underestimated [36]. This is probably because most of what they do is rarely measured, particularly in the LMIC health care settings where most measures of quality of care provided focus almost exclusively on more medical aspects of care [37, 38]. Therefore, measuring what nurses do and the quality of the care they deliver is essential in demonstrating the value of nurses and their work in promoting safety. These measurements will also be useful in highlighting the implications of workforce shortages and identifying opportunities for improving care whilst building improvement networks to promote nurse-led initiatives.

Our proposed set of indicators needs to be considered in light of the following limitations. Firstly, our review methods and stakeholder engagement differed from the more formal structured approaches of undertaking a systematic review and Delphi approach to indicator development. However, the process of developing and selection of indicators involved a wide range of stakeholders and were agreed upon through a consensus-based approach hence providing face validity. Although our final list of indicators (*n* = 52) have not formally been validated with a wider stakeholder group, we feel it provides an initial indicator set for testing in future studies of nursing care provision. We recognise that some indicators might be considered more critical than others such as those linked to patient outcomes (e.g. mortality) or due to their overall contribution to quality care. We adopted a simple approach giving each indicator equal weights that was deemed easiest for the diverse expert group to understand. The aim was to generate an initial set of indicators that can be further evaluated with the potential for introducing weighting based on further work. As such, this list is only indicative of what aspects of nursing should be measured and does not take into account the relative importance of various indicators. Secondly, anecdotal evidence and from the literature [39–42] suggests that documentation of nursing is often fragmented, completed on several forms, sometimes in triplicate, and often completed in free text. This may undermine the application of the proposed indicators that are based on document review. As such, piloting of the proposed indicators in routine practice to evaluate their feasibility, reliability and construct validity will be important. To monitor and track the proposed NSIs may require better tools to support nursing care documentation, for instance, structured nursing notes. Similar efforts of co-designing structured nursing forms in Uganda and the United Kingdom have shown improvements in communication between nurses and other professionals whilst reducing time spent on documentation [43, 44].

## Conclusion

Our proposed nurse-sensitive indicators informed by the literature and developed with stakeholders provide an opportunity for identifying gaps, developing targeted interventions for investment and improving care and mechanisms to support governance and accountability mechanisms that improve quality in LMIC health systems. The proposed NSIs for Kenya contribute to the dearth of information globally on NSI for monitoring quality of nursing care, particularly for LMICs. Further work on their validation through implementation, refinement and adaptation is required to generate a widely agreed set of standardised indicators. The latter provides an opportunity for LMICs to establish or join national or regional professional learning networks such as those in HICs [34, 45] or that are emerging in LMICs [46] that are showing success in achieving high-quality care through quality improvement and learning. Finally, measures of nursing quality might strengthen the voice of nurses in policy and practice and their position in planning and management roles where the nursing voice is often lacking.

## Data Availability

All data generated or analysed during this study are included in this published article.

## Abbreviations

HIC: High-income country
IPSG: International Patient Safety Goals
IQR: Inter-quartile range
JCI: Joint Commission International
LMIC: Low- and middle-income country
NSIs: Nurse-sensitive indicators
NQF: National Quality Forum
UNICEF: United Nations Children’s Fund
WHO: World Health Organization

## References

[1] Kruk ME, Gage AD, Arsenault C, Jordan K, Leslie HH, Roder-DeWan S, et al. High-quality health systems in the Sustainable Development Goals era: time for a revolution. Lancet Glob Heal [Internet]. 2018 Nov 1 [cited 2018 Dec 1];6(11):e1196–252. Available from: http://www.ncbi.nlm.nih.gov/pubmed/30196093.

[2] Griffiths P, Jones S, Maben J, Murrells T. State of the art metrics for nursing : a rapid appraisal. 2008;(October 2015):1–38.

[3] Redfern SJ, Norman IJ. Measuring the quality of nursing care: a consideration of different approaches. J Adv Nurs. 1990/11/01. 1990;15(11):1260–71. 10.1111/j.1365-2648.1990.tb01741.x 2269748

[4] Mueller C, Karon SL. ANA nurse sensitive quality indicators for long-term care facilities. J Nurs Care Qual. 2004/01/14. 2004;19(1):39–47. 10.1097/00001786-200401000-00009 14717147

[5] Chen L, Huang LH, Xing MY, Feng ZX, Shao LW, Zhang MY, et al. Using the Delphi method to develop nursing-sensitive quality indicators for the NICU. J Clin Nurs. 2016/07/13. 2017;26(3–4):502–13. 10.1111/jocn.13474 27404730

[6] Burston S, Chaboyer W, Gillespie B (2014). Nurse-sensitive indicators suitable to reflect nursing care quality: a review and discussion of issues. J Clin Nurs.

[7] National Quality Forum. National Voluntary Consensus Standards for Nursing-Sensitive Care: an initial performance measure set. 2004.

[8] Mccance T, Telford L, Wilson J, Macleod O, Dowd A (2012). Identifying key performance indicators for nursing and midwifery care using a consensus approach. J Clin Nurs.

[9] Peters MDJ, Godfrey CM, Khalil H, McInerney P, Parker D, Soares CB (2015). Guidance for conducting systematic scoping reviews. Int J Evid Based Healthc.

[10] Colquhoun HL, Levac D, O’Brien KK, Straus S, Tricco AC, Perrier L, et al. Scoping reviews: time for clarity in definition, methods, and reporting. J Clin Epidemiol [Internet]. 2014;67(12):1291–4. Available from: http://www.ncbi.nlm.nih.gov/pubmed/25034198. 10.1016/j.jclinepi.2014.03.013 25034198

[11] Tricco AC, Lillie E, Zarin W, O’Brien KK, Colquhoun H, Levac D, et al. PRISMA Extension for Scoping Reviews (PRISMA-ScR): checklist and explanation. Ann Intern Med [Internet]. 2018 Oct 2 [cited 2019 Jul 3];169(7):467. Available from: http://annals.org/article.aspx?doi = 10.7326/M18-0850. 10.7326/M18-0850 30178033

[12] National Quality Forum. Measure evaluation criteria [Internet]. [cited 2019 Jul 16]. Available from: http://www.qualityforum.org/Measuring_Performance/Submitting_Standards/Measure_Evaluation_Criteria.aspx.

[13] National Quality Forum. NQF: What we do [Internet]. [cited 2019 Aug 3]. Available from: http://www.qualityforum.org/what_we_do.aspx.

[14] Murphy GA V, Omondi GB, Gathara D, Abuya N, Mwachiro J, Kuria R, et al. Expectations for nursing care in newborn units in Kenya: moving from implicit to explicit standards. BMJ Glob Heal [Internet]. 2018 [cited 2018 Aug 28];3(2):e000645. Available from: http://www.ncbi.nlm.nih.gov/pubmed/29616146. 10.1136/bmjgh-2017-000645 PMC5875677 29616146

[15] Keene CM, Aluvaala J, Murphy GAV, Abuya N, Gathara D, English M. Developing recommendations for neonatal inpatient care service categories: Reflections from the research, policy and practice interface in Kenya. BMJ Glob Heal. 2019;4(2). 10.1136/bmjgh-2018-001195 PMC6441269 30997163

[16] Joint Commission International. International Patient Safety Goals | Joint Commission International [Internet]. [cited 2019 Jul 16]. Available from: https://www.jointcommissioninternational.org/improve/international-patient-safety-goals/.

[17] Joint Commission International. Who is JCI - who we are | Joint Commission International [Internet]. [cited 2019 Aug 3]. Available from: https://www.jointcommissioninternational.org/about-jci/who-is-jci/.

[18] Mark Foulkes. Nursing metrics: measuring quality in patient care. Nurs Stand. 2011;25(42):40–5. 10.7748/ns2011.06.25.42.40.c8582 21826870

[19] Maben J, Morrow E, Ball J, Robert G, Griffiths P. High quality care metrics for nursing. Natl Nurs Res Unit, King’s Coll London [Internet]. 2012;(August):1–53. Available from: http://eprints.soton.ac.uk/346019/1/High-Quality-Care-Metrics-for-Nursing%2D%2D%2D%2DNov-2012.pdf.

[20] Koy V, Yunibhand J, Angsuroch Y (2016). The quantitative measurement of nursing care quality: a systematic review of available instruments. Int Nurs Rev..

[21] Donabedian A. Evaluating the quality of medical care. Milbank Q [Internet]. 1966;44(No. 3, Pt. 2):166–203. Available from: http://www.pubmedcentral.nih.gov/articlerender.fcgi?artid=2690293&tool=pmcentrez&rendertype = abstract. 5338568

[22] Kunaviktikul, Anders, Chontawan, Nuntasupawat, Srisuphan, Pumarporn, et al. Development of indicators to assess the quality of nursing care in Thailand. Nurs Heal Sci. 2005/11/08. 2005;7(4):273–80. 10.1111/j.1442-2018.2005.00247.x 16271134

[23] Zhang Y, Liu L, Hu J, Zhang Y, Lu G, Li G, et al. Assessing nursing quality in paediatric intensive care units: a cross-sectional study in China. Nurs Crit Care. 2016:1–7. 10.1111/nicc.12246 27212426

[24] Langemo DK, Anderson J, Volden CM (2003). Nursing quality outcome indicators. JONA J Nurs Adm.

[25] MA C, PA H. The Nightingale metrics: nurses at one institution improved outcomes by putting patients “in the best condition for nature to act.”. Am J Nurs [Internet] 2006;106(10):66–70. Available from: http://search.ebscohost.com/login.aspx?direct=true&db=cin20&AN=106202997&site=ehost-live.

[26] Lacey SR, Klaus SF, Smith JB, Cox KS, Dunton NE. Developing measures of pediatric nursing quality. J Nurs Care Qual. 2006/07/04. 2006;21(3):210–2. 10.1097/00001786-200607000-00004 16816600

[27] Stratton KM. Pediatric nurse staffing and quality of care in the hospital setting. J Nurs Care Qual [Internet] 2008;23(2):105–114. Available from: http://content.wkhealth.com/linkback/openurl?sid=WKPTLP:landingpage&an=00001786-200804000-00003. 10.1097/01.NCQ.0000313758.33654.49 18344775

[28] Pazargadi M, Tafreshi MZ, Abedsaeedi Z, Majd HA, Lankshear AJ. Proposing indicators for the development of nursing care quality in Iran. Int Nurs Rev [Internet]. 2008 Dec [cited 2016 Nov 21];55(4):399–406. Available from: http://doi.wiley.com/10.1111/j.1466-7657.2008.00642.x. 10.1111/j.1466-7657.2008.00642.x 19146550

[29] Riehle AI, Hanold LS, Sprenger SL, Loeb JM. Specifying and standardizing performance measures for use at a national level: implications for nursing-sensitive care performance measures. Med Care Res Rev [Internet] 2007;64(2 suppl):64S-81S. Available from: http://mcr.sagepub.com/cgi/doi/10.1177/1077558707299263. 10.1177/1077558707299263 17406012

[30] Joint Learning Network for Universal Health Coverage [Internet]. [cited 2019 Aug 5]. Available from: http://www.jointlearningnetwork.org/.

[31] Safe Care: Basic healthcare standards [Internet]. [cited 2019 Aug 5]. Available from: https://www.safe-care.org/.

[32] The Council for Health Service Accreditation of Southern Africa (COHSASA) [Internet]. [cited 2019 Aug 5]. Available from: http://cohsasa.co.za/.

[33] Hospitals - Achieve Accreditation | Joint Commission International [Internet]. [cited 2019 Jul 16]. Available from: https://www.jointcommissioninternational.org/achieve-hospitals/.

[34] Montalvo I. The National Database of Nursing Quality Indicators (NDNQI). Online J Issues Nurs [Internet]. 2007;12(3):http://www.nursingworld.org/MainMenuCategories/ANA. Available from: http://search.ebscohost.com/login.aspx?direct=true&db=rzh&AN=2009867881&site=ehost-live.

[35] Ranegger R, Hackl WO, Ammenwerth E (2015). Development of the Austrian Nursing Minimum Data Set (NMDS-AT): the third Delphi round, a quantitative online survey. Stud Health Technol Inform.

[36] All-Party Parliamentary Group on Global Health. Triple impact: how developing nursing will improve health, promote gender equality and support economic growth. 2016.

[37] Gathara D, Nyamai R, Were F, Mogoa W, Karumbi J, Kihuba E, et al. Moving towards routine evaluation of quality of inpatient pediatric care in Kenya. PLoS One. 2015;10(3). 10.1371/journal.pone.0117048 PMC4378956 25822492

[38] Reyburn H, Mwakasungula E, Chonya S, Mtei F, Bygbjerg I, Poulsen A, et al. Clinical assessment and treatment in paediatric wards in the north-east of the United Republic of Tanzania. Bull World Heal Organ. 2008/02/26. 2008;86(2):132–9. 10.2471/BLT.07.041723 PMC2647389 18297168

[39] Hardey M, Payne S, Coleman P (2000). “Scraps”: hidden nursing information and its influence on the delivery of care. J Adv Nurs.

[40] Kebede M, Endris Y, Zegeye DT. Nursing care documentation practice: the unfinished task of nursing care in the University of Gondar Hospital. Informatics Heal Soc Care [Internet]. 2017;42(3):290–302. Available from: 10.1080/17538157.2016.1252766. 10.1080/17538157.2016.1252766 27918228

[41] Asamani JA, Amenorpe FD, Babanawo F, Ofei AMA. Nursing documentation of inpatient care in eastern Ghana. Br J Nurs [Internet] 2014;23(1):48–54. Available from: http://www.magonlinelibrary.com/doi/abs/10.12968/bjon.2014.23.1.48. 10.12968/bjon.2014.23.1.48 24406496

[42] Wang N, Hailey D, Yu P. Quality of nursing documentation and approaches to its evaluation: a mixed-method systematic review. J Adv Nurs [Internet]. 2011 1 [cited 2018 Mar 26];67(9):1858–75. Available from: http://doi.wiley.com/10.1111/j.1365-2648.2011.05634.x. 10.1111/j.1365-2648.2011.05634.x 21466578

[43] Chatterjee MT, Moon JC, Murphy R, McCrea D (2005). The “OBS” chart: an evidence based approach to re-design of the patient observation chart in a district general hospital setting. Postgrad Med J.

[44] Okaisu EM, Kalikwani F, Wanyana G, Coetzee M. Improving the quality of nursing documentation: an action research project. Curationis [Internet]. 2014 [cited 2020 Feb 13];37(2):E1-11. Available from: http://www.ncbi.nlm.nih.gov/pubmed/26864179. 10.4102/curationis.v37i2.1251 26864179

[45] Magnet Model [Internet]. [cited 2019 Jul 16]. Available from: https://www.nursingworld.org/organizational-programs/magnet/magnet-model/.

[46] Tuti T, Bitok M, Malla L, Paton C, Muinga N, Gathara D, et al. Improving documentation of clinical care within a clinical information network: an essential initial step in efforts to understand and improve care in Kenyan hospitals. BMJ Glob Heal [Internet]. 2016 [cited 2019 Jul 16];1(1):e000028. Available from: http://www.ncbi.nlm.nih.gov/pubmed/27398232. 10.1136/bmjgh-2016-000028 PMC4934599 27398232

